# Supplementary material for: Testing the individual and social learning abilities of task-naïve captive chimpanzees (Pan troglodytes sp.) in a nut-cracking task
Source: PeerJ. 2020 Mar 10;8:e8734. doi: 10.7717/peerj.8734 (PMC7069405; doi:10.7717/peerj.8734)
Supplement: Supplemental Information 6 [file peerj-08-8734-s006.docx]

| Term | Definition |
| --- | --- |
| Putative cultural trait | Those traits that have been identified to be potentially culturally dependent (CDT; see below), often through the method of exclusion (*sensu* Whiten et al., 1999). These traits have been shown to be customary or habitual in one geographically distinct community but absent in another (though not necessarily culturally distinct; see also below). However, these traits are yet to be subjected to the Latent Solutions (LS) testing methodology, and therefore cannot (yet) be considered truly culture dependent. |
| Culture Dependent Trait (CDT) | A culture dependent traits, or CDT, (*sensu* Reindl et al., 2017) is a behaviour and/or artefact (trait) that has been shown to be reliant on a copying variant of social learning (see below) in order for it to occur. These traits should not emerge and/or be maintained within culturally unconnected communities and/or within communities (and species) that lack copying variants of social learning. If these traits emerge in these cases then it is more parsimonious to assume that they are Latent Solutions (see below), though in the case of a mere lack of copying skill this claim would still need to be tested via the LS testing methodology. |
| Latent Solutions (LS) testing methodology | This is the methodology used to test the claim that a trait should be considered a CDT – where the alternative is that the behaviour is within the species’ Zone of Latent Solutions (ZLS; see below). This methodology involves exposing naïve, motivated subjects at the correct ontogenetic stage to the ecological conditions (i.e., raw materials) required to express the target trait and observing them for a given time period to determine if the trait reappears “from scratch” (Hedwig & Tennie, 2008). Species that ‘fail’ the LS test can have the target trait provisionally excluded from their ZLS (though the possibility remains that it could be added in at a later date, should it be observed in another culturally unconnected population). Therefore, to fully exclude a target behaviour from the species level ZLS several culturally distinct/independent populations should fail to express the target behaviour. Recent versions of the LS methodology differentiate between two ‘standards’, which allow researchers to include behaviours with a low relative probability of reoccurrence (e.g., comparatively complex traits such as nut cracking) in a species’ ZLS following just one reinnovation (single case standard; Bandini & Tennie 2018). |
| Latent Solution | These are traits within a species’ ZLS (Tennie et al., 2009), i.e., those traits that every member of that species has the capacity to reinnovate in the absence of social learning and enculturation by another species (Tennie et al. 2009; in press). However, it is important to note that outside of controlled testing conditions (e.g., LS tests) the vast majority (if not all) of these traits are socially influenced in their *frequency* across individuals, by non-copying variants of social learning (e.g., local enhancement). As a result, not every individual will at once show the trait. Equally importantly, not every member of a species will express their all LS their entire species’ ZLS capacity. The realised mix of LS expressed in a given individual is influenced by several factors, such as the individual’s physiological state (e.g., is it currently motivated to access a fatty substance such as a nut), access to non-copying social learning opportunities (i.e., if others around it engage in the latent solution), current circumstance (e.g., presence or absence of the raw materials required) etc. (see Tennie et al. in press for more details). |
| Zone of Latent Solutions (ZLS) | This is the potential repertoire of traits that any typically developing individual within a species has the potential ability to develop ‘from scratch’. It is influenced by a range of factors, including genetic predispositions and evolved cognitive skills (Tennie et al., 2009; in press). Importantly, not every individual within a species needs to express their species’ full ZLS range (see above) and therefore the ZLS of one individual may be ‘smaller’ than another of the same species and the same is true for single populations of the species (their realised mix of LS is often smaller than the full species ZLS). This is not to say that all species are restricted by their ZLS: a species that can copy traits can move beyond their ZLS (Tennie et al., 2009; Reindl et al., 2017). |
| Cumulative Culture | Cumulative culture is the variant of culture that a species capable of copying can develop. The human species has cumulative culture (in addition to our ZLS). Cumulative culture requires several conditions to be met:   - The ability to ‘ratchet up’ traits (Tomasello et al., 1993). The behaviour should show some evidence of cumulated, culturally inherited change (e.g., in complexity and/or in efficiency) across generations with limited ‘slippage’ across these generations. - Logically, the requirement of inheritance requires an inheritance mechanisms. Thus, cumulative culture must be underlain by copying variants of social learning – i.e. those that allow the inheritance of the form of the trait itself. There is still debate as for the concrete possibilities here, but most discussed are action and environmental results copying – including teaching of these types of information (Caldwell & Millen, 2008; Reindl et al., 2017; Tennie et al. 2009; in press). - Cumulative culture results in path-dependent cultural traits. Thus, the resultant traits should be beyond the capacity of a naïve individual to innovate within a single lifetime (Boyd & Richerson, 1996). Cumulative cultural traits should be outside the ZLS (though there may be a grey zone for some time at the beginning; Tennie et al. in press) |
| Social Learning | Social learning can be largely defined as the process of an individual being influenced by its perception of traits produced by others. This influence must alter the traits produced by the observer – the observer may then show a decrease or an increase in the frequency of a trait (non-copying variants of social learning) and/or the form of the trait (copying variants of social learning; compare Tennie et al. in press). There are myriads of ways in which social learning mechanisms are currently divided. Whiten et al. (2004) provide an overview of currently used terms. |
